# Supplementary material for: Pregnancy-induced maternal microchimerism shapes neurodevelopment and behavior in mice
Source: Nat Commun. 2022 Aug 5;13:4571. doi: 10.1038/s41467-022-32230-2 (PMC9356013; doi:10.1038/s41467-022-32230-2)
Supplement: Supplementary file 1 — Supplementary Information [file 41467_2022_32230_MOESM1_ESM.pdf]

Supplementary information

Pregnancy-induced maternal microchimerism shapes neurodevelopment and behavior in mice

Steven Schepanski[1,2], Mattia Chini[2], Veronika Sternemann[1,2], Christopher Urbschat[1], Kristin Thiele[1], Ting Sun[3,4], Yu Zhao[3], Mareike Poburski[1,2], Anna Woestemeier[5], Marie-Theres Thieme[1], Dimitra E. Zazara[1], Malik Alawi[6], Nicole Fischer[7], Joerg Heeren[8], Nikita Vladimirov[9], Andrew Woehler[9], Victor G. Puelles[10], Stefan Bonn[3], Nicola Gagliani[5], Ileana L. Hanganu-Opatz[2,‡,*] & Petra C. Arck[1,‡,*]

Affiliations:

[1] Division of Experimental Feto-Maternal Medicine, Department of Obstetrics and Fetal Medicine, University Medical Center Hamburg-Eppendorf, Hamburg, Germany

[2] Institute for Developmental Neurophysiology, Center for Molecular Neurobiology Hamburg, University Medical Center Hamburg-Eppendorf, Hamburg, Germany

[3] Institute of Medical Systems Biology, Center for Molecular Neurobiology Hamburg, University Medical Center Hamburg-Eppendorf, Hamburg, Germany

[4] Department of Neurogenetics, Max Planck Institute for Multidisciplinary Sciences, Göttingen, Germany

[5] Department of General, Visceral and Thoracic Surgery, University Medical Center Hamburg-Eppendorf, Hamburg, Germany

[6] Bioinformatics Service Facility, University Medical Center Hamburg-Eppendorf, Hamburg, Germany

[7] Institute of Medical Microbiology, Virology and Hygiene, University Medical Center Hamburg-Eppendorf, Hamburg, Germany

[8] Department of Biochemistry and Molecular Cell Biology, University Medical Center Hamburg-Eppendorf, Hamburg, Germany

[9] Berlin Institute for Medical Systems Biology, Max Delbrück Center for Molecular Medicine Berlin, Germany

[10] III Department of Medicine, University Medical Center Hamburg-Eppendorf, Hamburg, Germany

‡ Equal contribution; **Correspondence** should be addressed to P.C.A. (p.arck@uke.de) and I.L.H.-O (hangop@zmnh.uni-hamburg.de).

Supplementary Methods

Cell sorting

To FACS-purify MMc from wt offspring and fetal microglial cells of female offspring on E18.5, fetal microglial cells of one litter were stained with fluorochrome-conjugated antibodies against extracellular antigens under sterile conditions: anti-CD45.1 (1:400, BioLegend, Cat. No. 110705), anti-CD45.2 (1:100, BioLegend, Cat. No. 109823), anti-H-$2^{b/b}$ (1:20, BioLegend, Cat. No. 111507), anti-H-$2^{d/d}$ (1:200, BioLegend, Cat. No. 114712), anti-CD11b (1:200, BioLegend, Cat. No. 101241). Cell viability staining was performed using Zombie Yellow™ viability kit for 30 min at 4 °C on ice in the dark. Cells were sorted using the FACSAria III flow sorter (BD Life Sciences) with the Diva Software. For whole genome shotgun sequencing, per pooled litter $1x10^4$ microglial cells were obtained, sorted into Buffer RLT Plus (RNeasy micro Kit, Qiagen) containing 10 % 2-Mercaptoethanol (Sigma-Aldrich) and stored at -80 °C until further processing. For scRNA-seq, 16 fetal brains from 4 litters were pooled in order to collect $1x10^4$ MMc, which simultaneously minimized potential batch effects and integrates cells from 16 biological replicates.

Droplet-based single-cell RNA sequencing (scRNA-Seq)

Single cells were encapsulated in droplets using GemCode Technology and processed by using Chromium Single Cell 5' Reagent Kits (10X Genomics) following manufacturer's specifications. Briefly, transcriptome from each cell was captured in single droplet, and hybridized to gel beads with unique cell barcode and unique molecular identifier (UMI). Subsequently, barcoded cDNA was transcribed in droplets and then released for library construction. The constructed libraries were sequenced by HiSeq 4000 (Illumina) in paired-end to reach approximately 50.000 reads per cell[1].

ScRNA-seq data alignment and pre-processing

10x Genomics raw sequencing data were processed using CellRanger software (version 3.0.2, 10x Genomics, Pleasanton, CA) and the 10x mouse genome 10 3.0.0 release as the reference

(function cell ranger count). The matrices of cells and the unique molecular identifier (UMI) count were obtained and further processed by the R package Seurat (version 3.1.4)[2]. As a quality control (QC) step, we first filtered out genes detected in less than 3 cells and those cells in which less than 200 genes had non-zero counts. To remove potential doublets, cells with more than 5000 annotated genes were excluded. We further removed low-quality cells with more than 10 % mitochondrial genes of all detected genes.

We aimed to verify the purity of the sorted and sequenced MMc sample by scRNA-seq, however the utilized markers (CD45.1, CD45.2, H-2D$^{b/b}$, H-2D$^{d/d}$) had too small base pair differences. Also excluding fetal cells, contaminating the MMc sample by using paternal-specific SNP (Balb/c vs. C57BL/6) was not possible, due to limited sequencing depth.

Clustering analysis and cell population identification

The Seurat R package (version 3.1.4) was used to perform unsupervised clustering analysis on scRNA-seq data. Gene counts for cells that passed QC were first normalized by library size and log-transformed (function NormalizeData, normalization.method = "LogNormalize", scale.factor = 10000). Then, highly variable genes were detected (function FindVariableFeatures, selection.method = "vst", nfeatures = 2000). Principal component (PC) analysis was performed on the scaled data (function RunPCA, npcs = 30) in order to reduce dimensionality. The selected PCs were then used to compute the KNN graph based on the euclidean distance (function FindNeighbors), which later generated cell clusters using the function FindClusters. The resolution of FindClusters function was set to 0.4 by exploration of top marker genes of each cluster. Uniform Manifold Approximation and Projection (UMAP) was used to visualize clustering results. The top differentially expressed genes in each cluster were found using the FindAllMarkers function (min.pct = 0.25, logfc.threshold = 0.25) that ran Wilcoxon rank sum tests. The top expressed genes were then used to determine the cell population of each cluster. Differential gene expression analysis of each microglia

subpopulation within MMcs was computed by Model-based Analysis of Single-cell Transcriptomics (MAST) R package v.1.10.0[3].

RNA isolation and cDNA synthesis

FACS-purified microglia cells were stored at -20 °C until RNA isolation. Placentas were stored in RNA*later* at -20 °C. Tissue homogenization was done by using micro packaging vials with ceramic beads (1.4 mm) in a Precellys® 24 Tissue Homogenizer (Peqlab). Previously isolated PBMCs were stored at -20 °C until RNA isolation. RNA isolation and DNA digestion were carried out by using RNeasy Plus Universal Mini/Micro Kit (QIAGEN) and DNA-free Kit (Invitrogen by Thermo Fischer Scientific). cDNA synthesis was performed with random primers and SuperScript IV VILO (Invitrogen by Thermo Fischer Scientific). Concentration and purity of RNA and cDNA were assessed using the Multiskan Sky (ThermoScientific).

Quantitative real-time polymerase chain reaction (qRT-PCR)

Analyses of gene expression in placental tissue from MMc^{pos} and MMc^{low} offspring was performed using gene expression assays (Applied Biosystems by Thermo Fischer) for interferon beta 1 (*Ifn-y*, Mm01168134_m1), tumor necrosis factor (*Tnf-α*, Mm00443258_m1), and interleukin 6 (*Il-6*, Mm00446190_m1). Ubiquitin C (*Ubc*, Mm02525934_g1) and RNA polymerase II subunit A (*Polr2a*, Mm 00839502_m1) served as endogenous control to normalize cDNA content. Using a standard two-step qPCR protocol (initial 50 °C for 2 min and 95 °C for 10 min and 40 cycles: 15 s denaturation at 95 °C and 60 s annealing and extension at 60 °C), qPCR runs were performed in 10 µl reactions with the NanoQuant5 Real-Time PCR System (Applied Biosystems) and the corresponding software. Maternal PBMCs were evaluated for their cytokine and chemokine gene expression by RT^2 Profiler PCR Array (Cat. No. 330231, Qiagen) according to manufacturer's instructions. The fold change of *Rag2*$^{-/-}$*IL-2ryc*$^{-/-}$ over WT controls and MMc^{low} over MMc^{pos} was calculated employing the ΔΔCt method[4].

Leptin and adiponectin ELISA

Maternal leptin (R&D systems, Cat. No. DY498) and adiponectin (R&D systems, Cat. No. DY1119) levels were determined by ELISA according to the instructions of the manufacturer. For leptin, adult plasma samples were diluted in reagent dilution buffer 1:25. For adiponectin, adult plasma samples were diluted 1:10000, respectively.

Multiplex cytokine assay

Cytokine protein levels were quantified in previously stored plasma by a multiplex cytokine assay (Bio-Rad, Hercules, CA, Cat. No. M60009RDPD) in accordance with the manufacturer's instructions using the Luminex 200 system instrument (Luminex Corporation).

Pathogen detection

Samples were sent for diagnostic testing according to FELASA recommendations[5] by accredited laboratories (Biomedical Diagnostics, Hannover, Germany and GIM Gesellschaft fuer innovative Mikrooekologie mbH, Michendorf, Brandenburg, Germany). The screening for specific subset of mouse pathogens was performed after cultivation, GRAM staining and selective media by Matrix Assisted Laser Desorption Ionization — Time of Flight Mass Spectrometry (MALDI-TOF) or by PCR. Infections that are routinely detected by Immune fluorescents assay (IFA) will be analyzed also by ELISA. Parasitic pathogens can be detected via direct preparation, flotation and light microscope analyses respectively by PCR.

Imaging and image analysis

Slices were imaged with a confocal microscope (Leica DMI6000, Leica Microsystems). For Iba-1, Vglut1-, and Vglut2-staining, microscopic stacks were acquired as 1024x1024 pixels images with 750 nm Z-steps capturing 8 microglial cells within the mPFC and CA1 region of the HP, using a 63X objective. The overlay of Iba-1$^+$ and Vglut$^+$ signals was assessed using a previously published protocol[6,7] with Matlab and ImageJ. Microglia numbers and labeled synapses (Vglut1, Vglut2) were determined using the particle analyzer plugin for the ImageJ

software. The threshold for all signals was set to acquire optimal representation and kept constant during image analyses. Afterwards, the number was normalized to mm^2.

Co-cultures labelled by IHC were imaged with the Leica TCS SP5 confocal microscope and Leica application suite software (LAS-AF-lite). For Sholl analysis, microscopic stacks of three neurons per well were acquired as 3080x3080 pixel imaged in 710 nm Z-steps, using a 25X objective. For detections of neuron and microglia numbers, fluorescent signals were counted using photomicrographs acquired as described above. Sholl analysis was performed using ImageJ (https://imagej.net/plugins/sholl-analysis). Neuronal soma sizes were measured and the center specified. The semi-automatical "Simple Neurite Tracer" plugin was used to trace neuronal branches starting from the soma center. The resulting paths were used to generate a skeletonized picture of the branches. The intersections per concentric circle were used for further analysis.

Optical clearing and light-sheet microscopy

Optical clearing was performed using an adapted protocol from[8]. Briefly, intact brains were washed in PBS for 24 h, and were then incubated in CUBIC-L (TCI; Cat. No. T3740) for 24 h, followed was a 12 h wash in PBS and finally incubation in CUBIC-R+ (TCI; Cat. No. T3741) until translucency was achieved (approx. 12hrs).

The cleared samples were imaged with a custom-made light-sheet microscope mesoSPIM[9]. Cleared brains were immersed in CUBIC-R+ inside a small quartz cuvette (Portmann Instruments AG, UQ-205), which was placed inside a larger chamber (Portmann Instruments AG, Q754) filled with RI-matching oil (Cargille 19569). The light-sheet illumination was delivered sequentially from left and right side, resulting two different stacks, which were registered and fused into a single stack using BigStitcher software[10], using an iterative closest point registration algorithm, for each channel. The excitation laser lines (Hübner Photonics C-Flex: 405, 488, 561 nm) were used with corresponding detection filters (Chroma ET480/40m, ET525/50m, ET590/50m). The samples were imaged at low

magnification, filling the entire field of view of the objective (Olympus 1x MVPLAPO1x, zoom settings 1.6x). Images were post-processed with Imaris v9.6 (Bitplane). Representative images of tdTomato^{+} cells are shown in 3D and 2D in the developmental mouse brain.

RNA sequencing and bioinformatic analysis

After isolation, the RNA integrity was analyzed with the RNA 6000 Pico Chip on an Agilent 2100 Bioanalyzer (Agilent Technologies). RNA-Seq libraries were generated using the SMART-Seq v4 Ultra Low Input RNA Kit (Clontech Laboratories) as per the manufacturer's recommendations. From cDNA, final libraries were generated utilizing the Nextera XT DNA Library Preparation Kit (Illumina). Concentrations of the final libraries were measured with a Qubit 2.0 Fluorometer (Thermo Fischer Scientific) and fragment lengths distribution was analyzed with the DNA High Sensitivity Chip on an Agilent 2100 Bioanalyzer (Agilent Technologies). All samples were normalized to 2nM and pooled equimolar. The library pool was sequenced on the NextSeq500 (Illumina) with 1x75 bp, with approximately 20 Mio reads per sample. Sequence reads were aligned to the mouse reference assembly (GRCm38.98) with STAR (v.2.7.2d)[11]. Normalization and differential expression analysis were carried out with DESeq2[12]. Genes were considered to be differentially expressed if an absolute log2 fold change greater than 1 and an FDR smaller than 0.05 were observed.

Microbiome analysis

16S rRNA amplicon *library preparation, MiSeq sequencing, and bioinformatic analysis*. V3–V4 region 16S rRNA amplicons were generated using degenerate primers that contain the Illumina adapter consensus sequence F: (5'-TCGTCGGCAGCGTCAGATGTGTATAAGAGACA-GCCTACGGGNGGCWGCAG-3') and R: (5'-GTCTCGTGGGCTCGGAGATGTGTAT-AAGAGACAGGACTACHVGGGTATCTAATCC-3`) as previously published[13-15]. Samples were multiplexed using the Illumina Nextera XT Index Kit to construct barcoded libraries. Subsequent libraries were multiplexed and sequenced by 500PE sequencing on the MiSeq

platform. FastQC (Babraham Bioinformatics, Babraham Institute, UK) was used to determine the average quality scores of each sample before and after paired reads. The paired ends in each sample were joined, and all sequences less than 250 bp and/or with a Phred score <33 were discarded. Quality filtering was applied using QIIME 53 (at Phred $\geq$ Q20). We performed operational taxonomic unit (OTU) clustering and alpha- and beta-diversity analysis using QIIME version 1.7. A chimera filter was applied using USEARCH 8.1. All sequences were clustered based on 97% similarity to reference sequences. The reads that did not meet the 97% similarity criteria were clustered de novo. Taxonomy levels of representative sequences in the OTU table were assigned at 95 % similarity based on the SILVA database. We calculated alpha diversity based on the Shannon diversity index. Analysis of beta diversity statistics (analysis of similarities, ANOSIM) was performed to determine if differences between the distributions of microbiota profiles from the five datasets were significant.

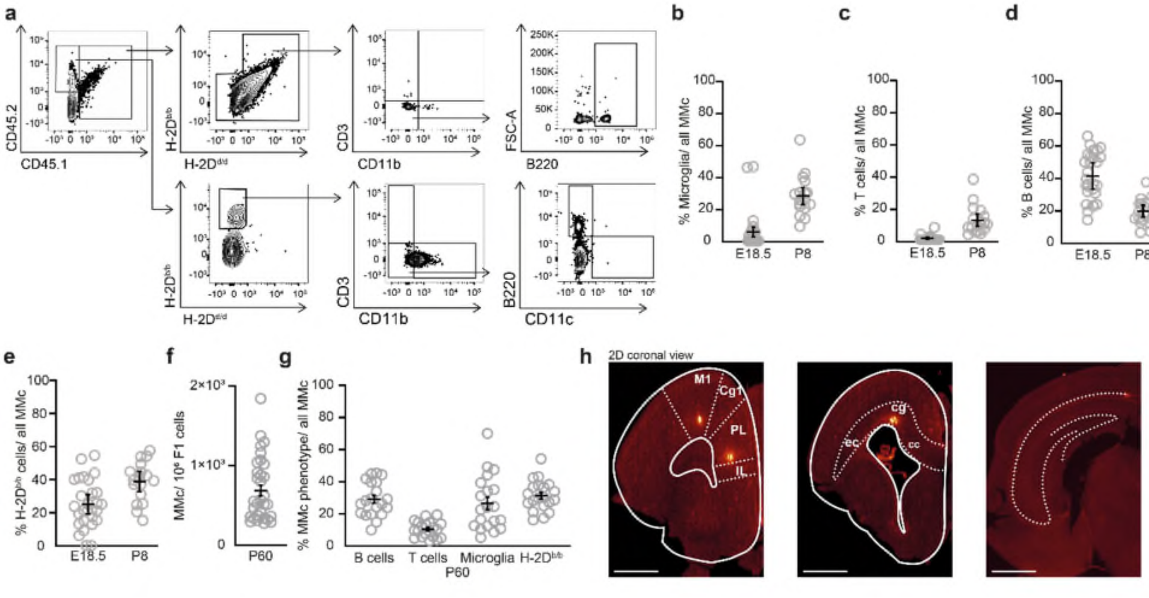

Supplementary Fig. 1. Cellular composition of MMc over the lifespan. **a**, Strategy to gate the enriched population of MMc at E18.5 in wt offspring. Cells were gated for singlets, followed by being gated for living cells (fixable viability dye, Zombie Yellow^{TM}), CD45.1/2^{+}, H-2D$^{b/d}$ (defined as fetal cells, upper path), and CD45.2^{+}, H-2D$^{b/b}$ (defined as MMc, lower path). Both populations were further phenotyped to identify immune cell subpopulations (CD3^{+}, CD11b^{+}, B220^{+}, and CD11c^{+}). **b-e**, Percentage of phenotypic subsets within MMc, identified by flow cytometry among brain cells at E18.5 (Analysis of microglia *n*=29; of T cells *n*=29; of B cells *n*=27; of H-2D$^{b/b}$ cells *n*=28) and P8 (Analysis of microglia *n*=17; of T cells *n*=17; of B cells *n*=17; of H-2D$^{b/b}$ cells *n*=17). **f**, Number of MMc identified by flow cytometry among brain cells from wt offspring isolated at P60 (*n*=31)**. g**, Percentage of phenotypic subsets within MMc in brains of wt offspring at P60 (Analysis of microglia *n*=20; of T cells *n*=20; of B cells *n*=20; of H-2D$^{b/b}$ cells *n*=20). **h**, 2D coronal view of prefrontal cortex subdivided into primary motor cortex (M1), cingulate cortex area 1 (Cg1), prelimbic cortex (PL), and infralimbic cortex (IL) (left). Coronal view subdivided into external capsule (ec), cingulum (cg), and corpus callosum (cc) (middle). Noteworthy, tdTomato^{+} MMc can be found in the cerebral vasculature lumen. Coronal view of hippocampus subdivided (right). Scale bar, 200 µm. 2D coronal sections were generated from wt offspring of one litter. Data are displayed as mean ± SEM and each circle denotes one pup.

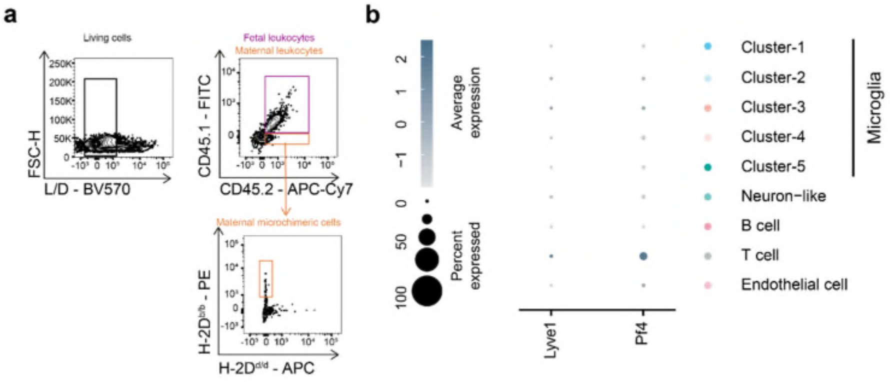

Supplementary Fig. 2. Molecular characterization of brain immune cells. a, Strategy for MMc isolation before scRNA-sequencing. After gating of living cells, MMc were enriched by $CD45.2^{+}$ and then $H\text{-}2D^{b/b}$. **b**, Gene expression of platelet factor 4 (*Pf4)* and lymphatic vessel endothelial hyaluronan receptor 1 (*Lyve1)* to exclude macrophage contamination in the microglia cluster. The dot size represents percentage of expression and the color represents its average expression within identified MMc clusters.

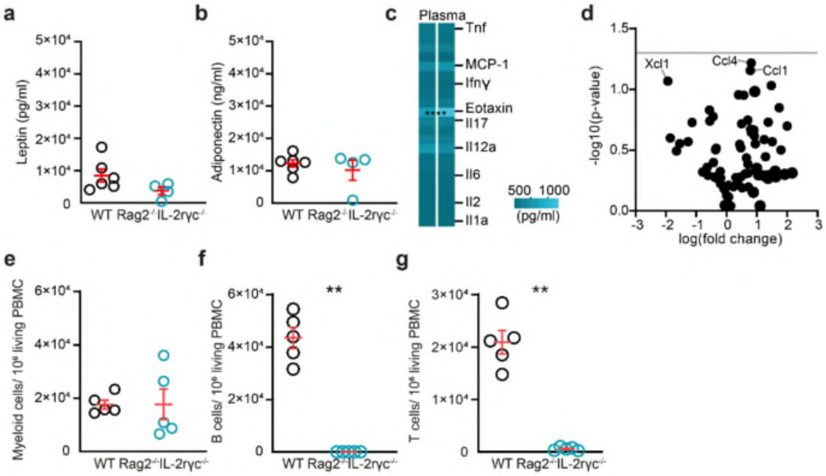

Supplementary Fig. 3. Maternal inflammatory profile. Plasma levels of peptide hormones (**a**) leptin (wt *n*=6, Rag2$^{-/-}$IL-2rγc$^{-/-}$ *n*=4) and (**b**) adiponectin (wt *n*=6, Rag2$^{-/-}$IL-2rγc$^{-/-}$ *n*=4) in dams at E18.5. **c**, Heatmap of cytokines and chemokines on the protein levels in maternal plasma of dams at E18.5, the lowest concentration is coded by the darkest color. **d**, Volcano plot depicting comparative gene expression changes of 84 cytokines and chemokines in maternal PBMCs at E18.5. Genes below the horizontal line show no significant change between wt and *Rag2$^{-/-}$IL-2rγc$^{-/-}$* dams. **e-g**, Numbers of peripheral immune cells of wt (*n*=5) and *Rag2$^{-/-}$IL-2rγc$^{-/-}$* (*n*=5) dams at E18.5. Significance was determined by a two-sided Mann-Whitney U test, $^{**}p < 0.01$. Data in **a**, **b**, **e-g** are displayed as mean ± SEM and each circle denotes one dam.

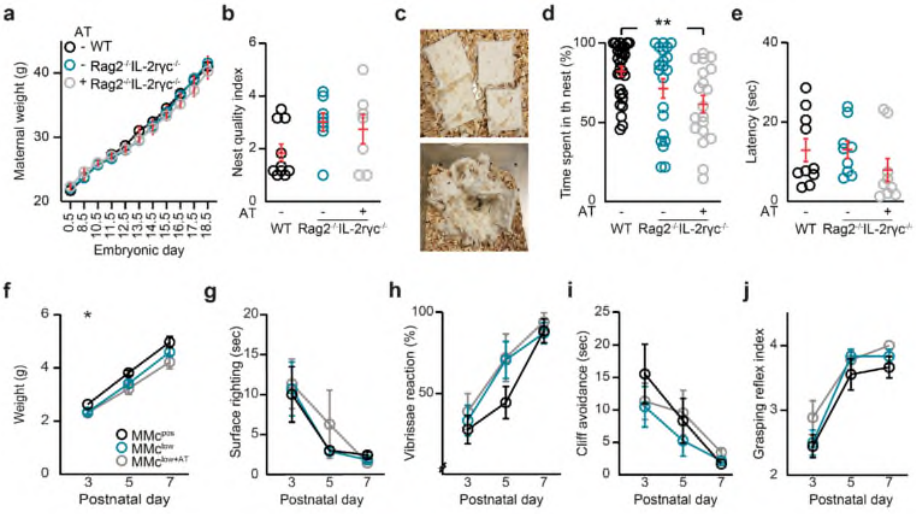

Supplementary Fig. 4. Perinatal parameter and offspring's early development. a, Maternal weight gain during pregnancy (wt *n*=12, *Rag2*$^{-/-}$*IL-2rγc*$^{-/-}$ *n*=12, *Rag2*$^{-/-}$*IL-2rγc*$^{-/-+AT}$ *n*=6). Of note, the data presented here also includes the group of *Rag2*$^{-/-}$*IL-2rγc*$^{-/-}$pregnant females receiving immune cells via adoptive transfer, as introduced in **Fig. 5a**. **b**, Nest building quality of wt (*n*=10), *Rag2*$^{-/-}$*IL-2rγc*$^{-/}$ (*n*=8)$^{-}$, and *Rag2*$^{-/-}$*IL-2rγc*$^{-/-}$ dams upon adoptive transfer (*n*=7). **c**, Representative photographs of a nest scored with 0 points (top) and 5 points (bottom) quality index. **d**, Postnatal maternal caring behavior, quantified as maternal time spent on vs. off the nest with P2, P4, and P6 pups over 60 minutes of observation (wt *n*=29, *Rag2*$^{-/-}$*IL-2rγc*$^{-/-}$ *n*=21, *Rag2*$^{-/-}$*IL-2rγc*$^{-/-+AT}$ *n*=19). **e**, Time latency until first pup is approached from a litter removed from the nest at P6 (wt *n*=10, *Rag2*$^{-/-}$*IL-2rγc*$^{-/-}$ *n*=9, *Rag2*$^{-/-}$*IL-2rγc*$^{-/-+AT}$ *n*=9). **f-j**, Quantification of weight (MMc^{pos} *n*=12, MMc^{low} *n*=9, MMc^{low+AT} *n*=9) and developmental milestones of MMc^{pos} (P3 *n*=9, P5 *n*=9, P7 *n*=9), MMc^{low} (P3 *n*=12, P5 *n*=12, P7 *n*=12), and MMc^{low+AT} (P3 *n*=9, P5 *n*=9, P7 *n*=9) offspring (as depicted in **Fig. 5**) during the first postnatal week. Significance in **a** and **f-j** was determined by a two-way ANOVA, $*p < 0.05$. Significance in **b**, **d**, and **e** was determined by a Kruskal-Wallis one-way ANOVA followed by Dunn's multiple comparison test, $**p < 0.01$. Data shown in **a**, **b**, **d-j** are shown as mean ± SEM, each circle shown in **a**, **b**, **d**, **e** corresponds to one dam, or to the mean of one litter in **f-j**.

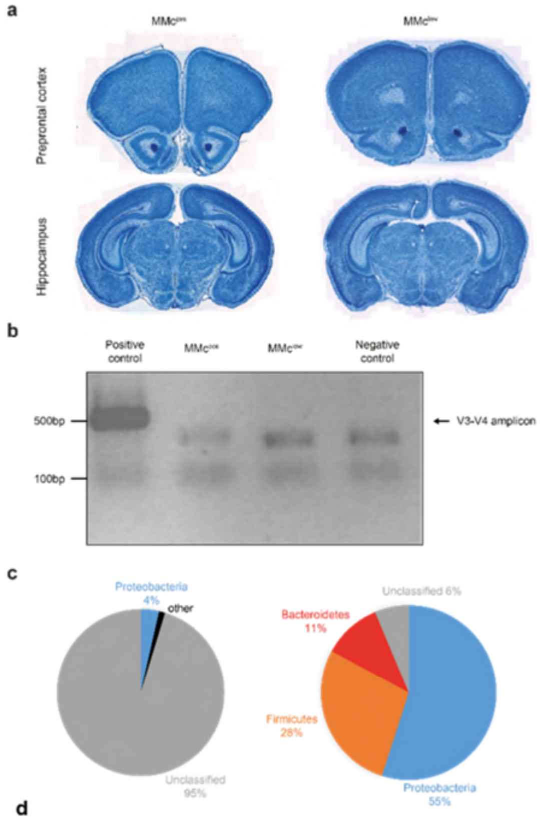

d

	Proteobacteria (%)	Firmicutes (%)	Bacteroidetes (%)	Other (%)	Unclassified (%)
Negative control	3.54	0	0	1.17	95.29
MMc^{pos}	2.4	0	0	0.2	97.4
MMc^{low}	4.12	0	0	0.9	94.98
Positive control	54.97	27.92	10.82	0	6.29

Supplementary Fig. 5. Microbial invasion of offspring's brains. a, Medial prefrontal cortex and hippocampal brain sections from MMc^{low} and MMc^{pos} offspring (*n*=1 each) were used for Gram staining, individual images of the tissue section were merge into a photomontage. **b**, V3–V4 region 16S rRNA amplicons were generated using previously published degenerate primers that contain the Illumina adapter consensus sequence[16]. Amplicons are shown by agarose gel electrophoreses. **c**, Amplicon sequencing results of the control samples using the Illumina Nextera XT Index kit and sequenced by 2x300bp paired end sequencing on the MiSeq platform. Pie charts represent from the left to the right the negative control, water sample showing less than 5% bacterial reads with most of them representing environmental proteobacteria commonly detected, even in negative and water controls and the taxonomic classification (phylum) of the positive control, artificial bacterial community. **d,** Taxonomic assignment of all sequencing results.

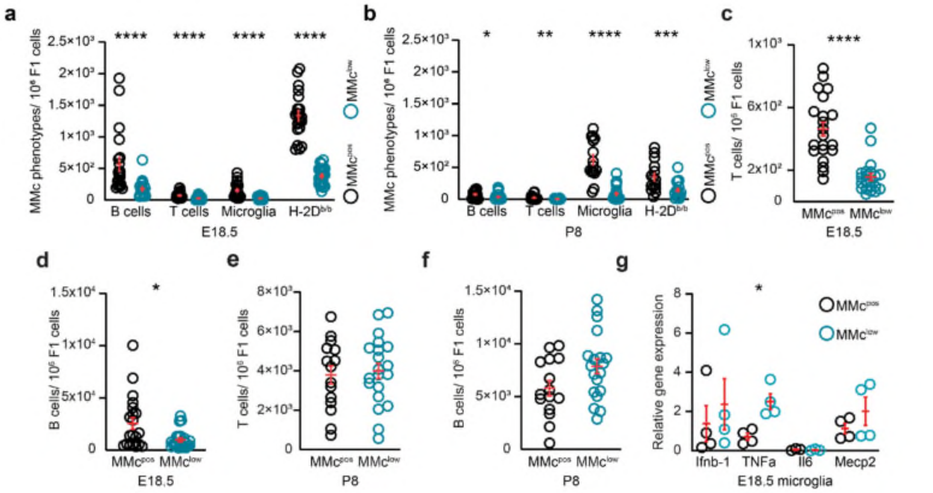

Supplementary Fig. 6. Characterization of brain immune cells. Number of MMc phenotypes identified by flow cytometry among brain cells from MMc^{pos} and MMc^{low} offspring isolated at E18.5 (**a**; analysis of B cells, MMc^{pos} *n*=21, MMc^{low} *n*=21; of T cells, MMc^{pos} *n*=21, MMc^{low} *n*=23; of microglia, MMc^{pos} *n*=21, MMc^{low} *n*=23; of H-2D$^{b/b}$ cells, MMc^{pos} *n*=21, MMc^{low} *n*=20) and at P8 (**b**; Analysis of B cells, MMc^{pos} *n*=16, MMc^{low} *n*=22; of T cells, MMc^{pos} *n*=16, MMc^{low} *n*=22; of microglia, MMc^{pos} *n*=15, MMc^{low} *n*=22; of H-2D$^{b/b}$ cells, MMc^{pos} *n*=15, MMc^{low} *n*=22). Number of T and B cells in the brain from MMc^{pos} and MMc^{low} offspring isolated at E18.5 and identified by flow cytometry (**c**; Analysis of T cells, MMc^{pos} *n*=21, MMc^{low} *n*=21; **d**; Analysis of B cells, same *n* as in **c** for MMc^{pos} and MMc^{low} offspring); same as **c,d** at P8 (Analysis of T cells, MMc^{pos} *n*=15, MMc^{low} *n*=20; of B cells, MMc^{pos} *n*=15, MMc^{low} *n*=20). **g**, Relative gene expression levels of genes responsible for microglia activity in FACS-purified microglia isolated from MMc^{pos} and MMc^{low} offspring at E18.5: Interferon β (*Ifnb-1*; MMc^{pos} *n*=4, MMc^{low} *n*=4), tumor necrosis factor alpha (*Tnfα*; MMc^{pos} *n*=4, MMc^{low} *n*=4), interleukin-6 (*Il6*; MMc^{pos} *n*=3, MMc^{low} *n*=3) and methyl CpG binding protein 2 (*Mecp2*; MMc^{pos} *n*=4, MMc^{low} *n*=4). The fold change was calculated over β-actin (*Actb*) and peptidylprolyl isomerase A (*Ppia*) expression using the ΔΔCt method. Significance was determined by a two-sided Mann-Whitney U test, *$p < 0.05$, **$p < 0.01$, ***$p < 0.001$, ****$p < 0.0001$. Data are displayed as mean ± SEM and each circle corresponds to one pup.

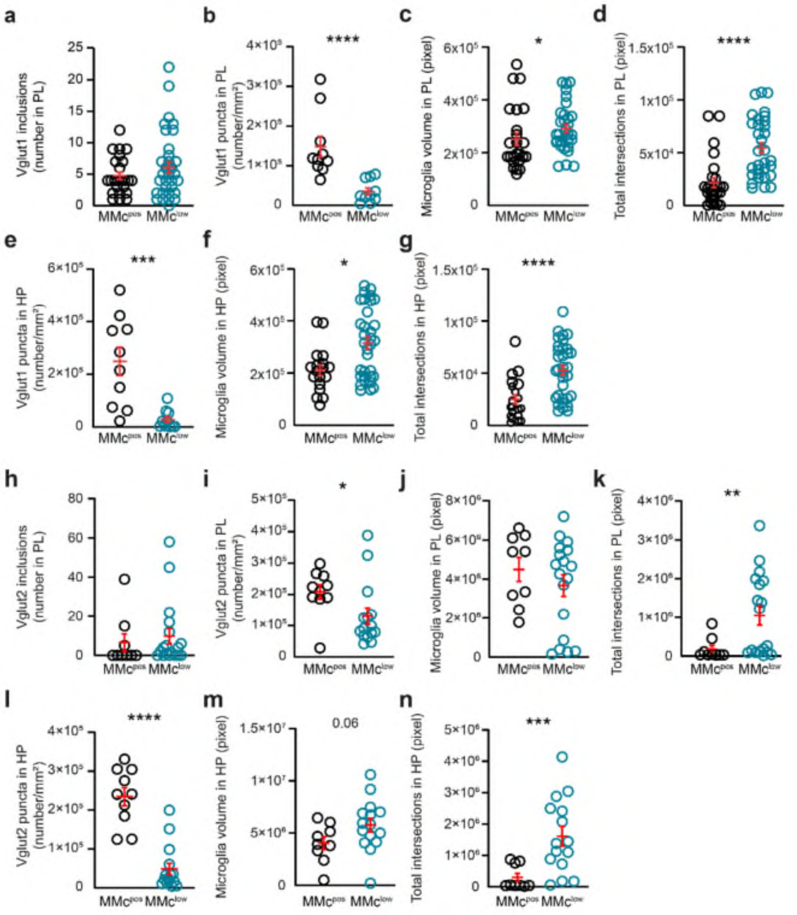

Supplementary Fig. 7. Characterization of MMc-mediated microglia engulfment. a-d, Vesicular glutamate transporter (Vglut)1^{+} synapse inclusion (MMc^{pos} n=24, MMc^{low} n=34), Vglut1^{+} synapse quantification (MMc^{pos} n=10, MMc^{low} n=10), microglia volume (MMc^{pos} n=24, MMc^{low} n=32) and total number of intersections (MMc^{pos} n=26, MMc^{low} n=32) in the PL of MMc^{pos} vs. MMc^{low} offspring at P8. **e-g**, same as **b-d** but for the HP (Synapses MMc^{pos} n=10, MMc^{low} n=10; Microglia volume MMc^{pos} n=18, MMc^{low} n=33; Total intersections MMc^{pos} n=20, MMc^{low} n=33). **h-n,** same as **a-g** but for Vglut2 expression (PL: Inclusions MMc^{pos} n=9, MMc^{low} n=18; Synapses MMc^{pos} n=10, MMc^{low} n=15; Microglia volume MMc^{pos} n=9, MMc^{low} n=18; Total intersections MMc^{pos} n=9, MMc^{low} n=18; HP: Synapses MMc^{pos} n=10, MMc^{low} n=15; Microglia volume MMc^{pos} n=9, MMc^{low} n=15; Total intersections MMc^{pos} n=9, MMc^{low} n=15). Significance was determined by a two-sided Mann-Whitney U test, *$p < 0.05$, **$p < 0.01$, ***$p < 0.001$, ****$p < 0.0001$. Data are displayed as mean ± SEM and each circle corresponds to one pup.

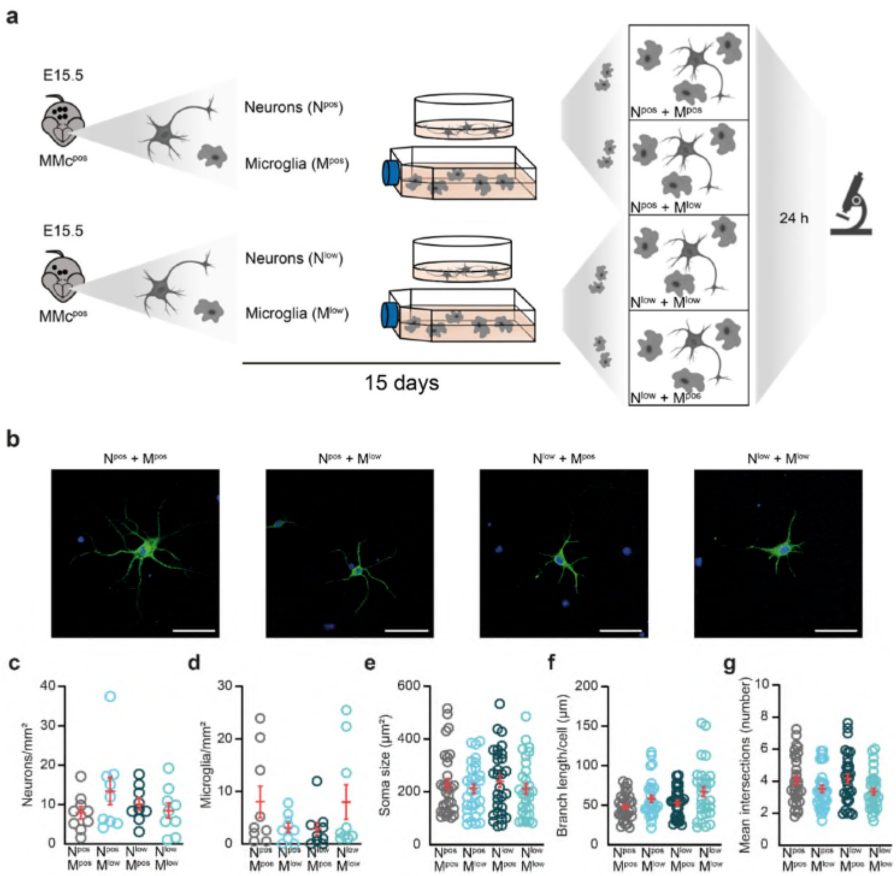

Supplementary Fig. 8. In vitro MMc^{low} microglia activity. **a**, Schematic illustration of *in vitro* set-up. **b**, Representative photomicrograph of neurons (green) with nuclei (blue) in different neuron and microglia co-culture combinations isolated from MMc^{pos} and MMc^{low} offspring. Scale bar, 50 µm. Photomicrographs were generated from three independently repeated experiments. **c-d**, Number of neurons (N^{pos}/M^{pos} *n*=9, N^{pos}/M^{low} *n*=9, N^{low}/M^{pos} *n*=9, N^{low}/M^{low} *n*=9) and microglia (N^{pos}/M^{pos} *n*=9, N^{pos}/M^{low} *n*=9, N^{low}/M^{pos} *n*=9, N^{low}/M^{low} *n*=9) quantified in different co-culture combinations. Significance in b was determined by the Friedman test followed by Dunn's multiple comparison test. **e-g**, Soma size (N^{pos}/M^{pos} *n*=30, N^{pos}/M^{low} *n*=30, N^{low}/M^{pos} *n*=30, N^{low}/M^{low} *n*=30), length of neuronal branches (N^{pos}/M^{pos} *n*=30, N^{pos}/M^{low} *n*=30, N^{low}/M^{pos} *n*=30, N^{low}/M^{low} *n*=30) and intersection (N^{pos}/M^{pos} *n*=30, N^{pos}/M^{low} *n*=30, N^{low}/M^{pos} *n*=30, N^{low}/M^{low} *n*=30) quantified as a mean per cell of different neuron-microglia mix co-culture. Significance was determined by a Kruskal-Wallis one-way ANOVA followed by Dunn's multiple comparison test. Data in **c-g** are displayed as mean ± SEM and each circle corresponds to one cell.

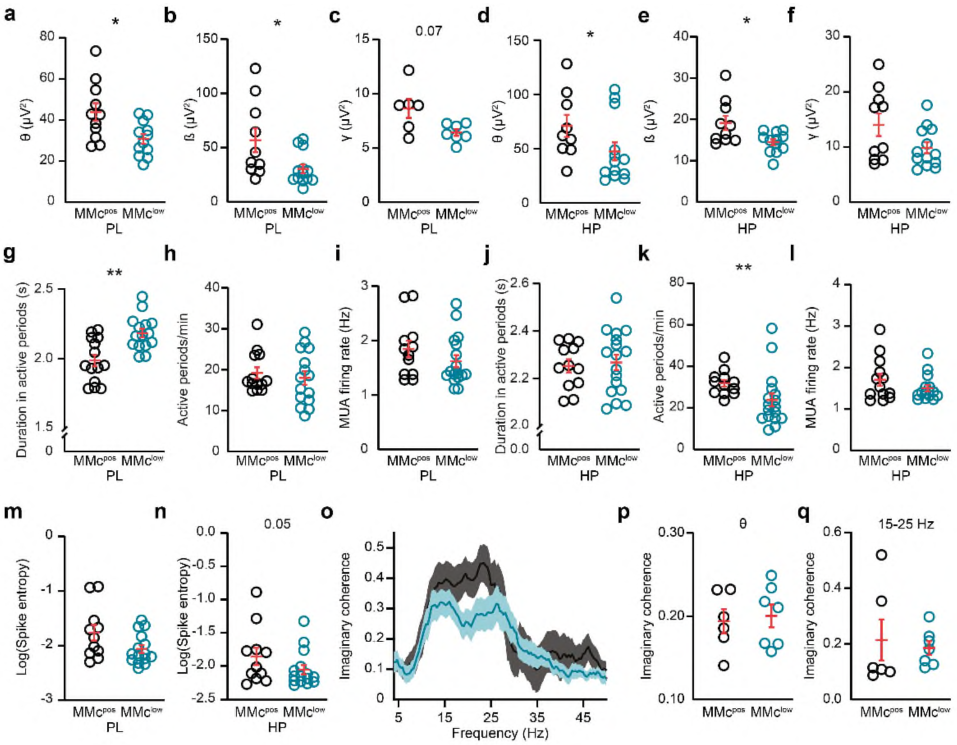

Supplementary Fig. 9. Properties of network oscillations and coupling in offspring with reduced MMc. Scatter plots displaying **a-c**, θ (4-12 Hz, MMc^{pos} n=11; MMc^{low} n=12), β (12-30 Hz, MMc^{pos} n=10; MMc^{low} n=12), and γ power (30-100 Hz, MMc^{pos} n=11; MMc^{low} n=11) quantified in prelimbic cortex (PL) of the medial prefrontal cortex in newborn mice (P8). **d-f**, same as **a-c** but for hippocampus (HP: θ, MMc^{pos} n=14; MMc^{low} n=15; β, MMc^{pos} n=13; MMc^{low} n=15; γ, MMc^{pos} n=13; MMc^{low} n=15). **g**, Duration of active periods (MMc^{pos} n=11; MMc^{low} n=15), **h**, occurrence of discontinuous oscillatory events (MMc^{pos} n=9; MMc^{low} n=13), and **i**, multi-unit activity (MUA, MMc^{pos} n=10; MMc^{low} n=13) quantified in the PL of newborn mice (P8). **j-l**, same as **g-i** but for HP (Duration in active periods: MMc^{pos} n=10; MMc^{low} n=13; Occurrence of discontinuous oscillatory events: MMc^{pos} n=12; MMc^{low} n=17; MUA: MMc^{pos} n=12; MMc^{low} n=17). **m**, Log transformed spike entropy quantified in the PL of newborn mice (P8, MMc^{pos} n=12; MMc^{low} n=17). **n**, same as **m** but for HP (MMc^{pos} n=11; MMc^{low} n=14). **o**, Imaginary coherence spectra for oscillatory activity simultaneously recorded in the PL and hippocampal CA1 area averaged for MMc^{pos} and MMc^{low} mice. **p-q**, Averaged imaginary coherence within 4-12 Hz and 15-25 Hz ranges in MMc^{pos} (n=6) and MMc^{low} (n=7) mice. Significance was determined by a two-sided Mann-Whitney U test, $*p < 0.05$, $**p < 0.01$. Data in o are displayed as median ± 1.5 IQR. Data are displayed as mean ± SEM and each circle denotes one pup.

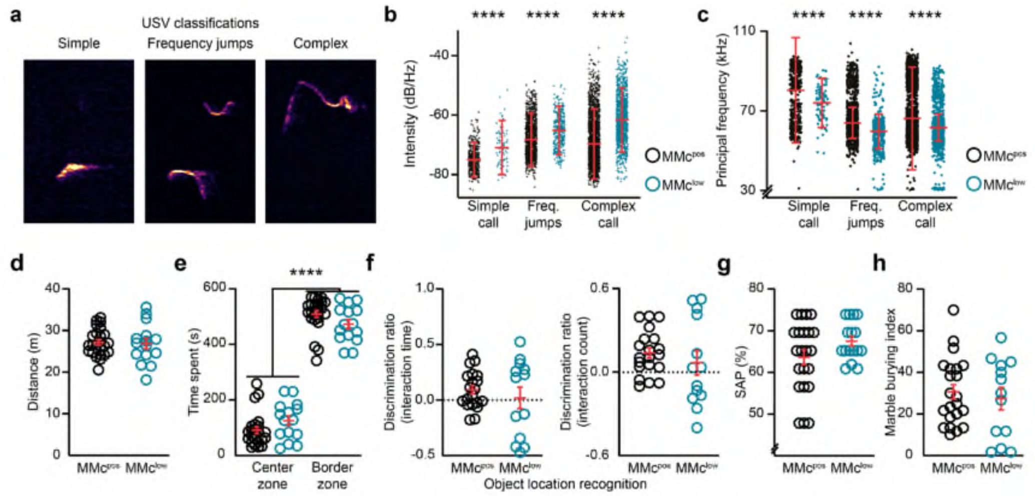

Supplementary Fig. 10. Behavioral assessments in offspring. **a**, Representative spectrogram of the three classified ultrasonic vocalizations (USVs) generated from one pup. **b-c**, Intensity and principal frequency of syllables emitted by MMc^{pos} (*n*=11) and MMc^{low} (*n*=8) pups at P8. USV are specified for simple calls, frequency jumps, complex calls, as recorded over a period of 10 minutes. Significance was determined by the Friedman test followed by Dunn's multiple comparison test, ****$p < 0.0001$. **d**, Distance travelled while performing an open field task (MMc^{pos} *n*=24; MMc^{low} *n*=15) and **e**, time spent in predefined center and border zone for MMc^{pos} (*n*=24) compared to MMc^{low} (*n*=15). **f**, Discrimination ratio of the time of interaction and the count of interactions with a newly located object for MMc^{pos} (left, *n*=20; right, *n*=20) and MMc^{low} (left, *n*=13; right, *n*=13). **g**, Scatter plot of spontaneous alternation pattern (SAP) of the first 25 Y-maze arm entries of MMc^{pos} (*n*=22) and MMc^{low} (*n*=16) offspring. **h**, Marble burying index showing the repetitive-like behavior between MMc^{pos} (*n*=22) and MMc^{low} (*n*=13). Significance in **d** and **f-h** was determined by a two-sided Mann-Whitney U test. Significance in e was determined by a two-way ANOVA followed by Dunn–Šidák's multiple comparison test, ****$p < 0.0001$. Data in b-h are displayed as mean ± SEM and each circle corresponds to one call or one pup.

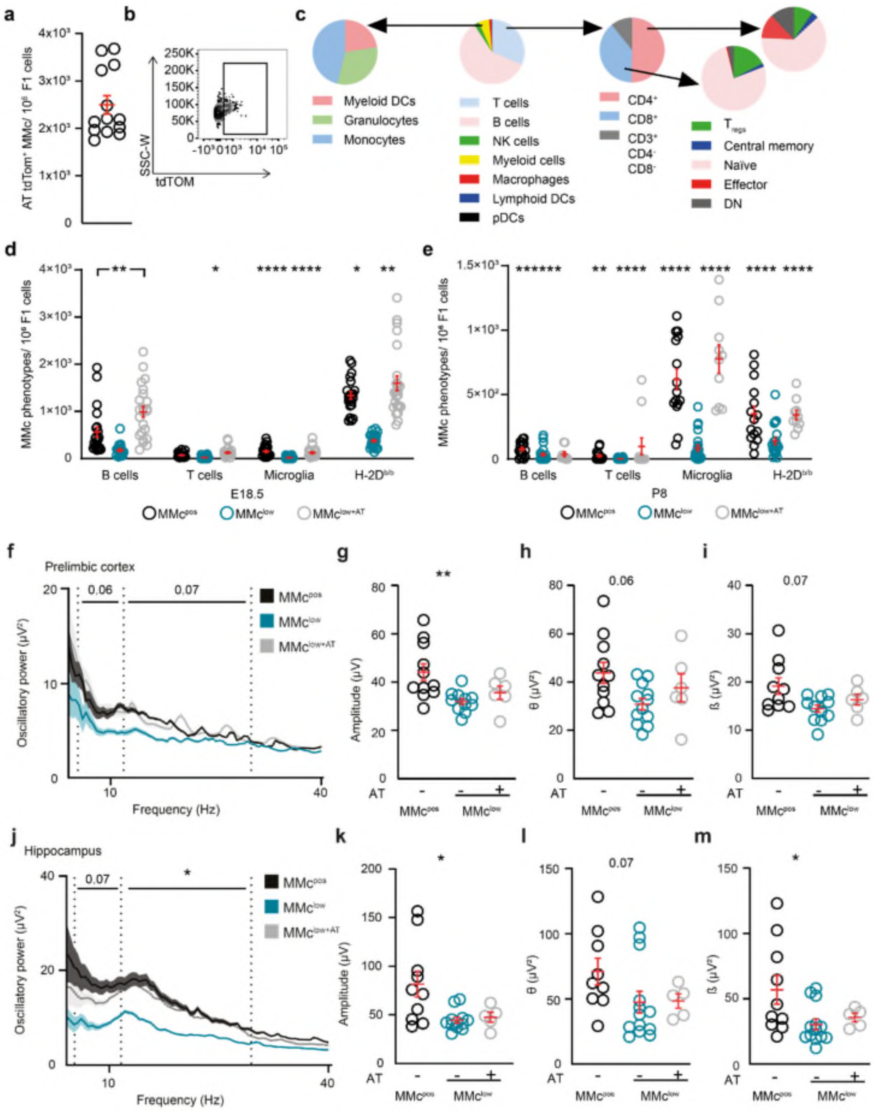

Supplementary Fig. 11. MMc effects on the offspring's brain after adoptive transfer (AT) of maternal immune cells. a, Numbers of tdTomato-expressing MMc detected in the wt offspring's brain (*n*=13) following AT of tdTomato-expressing immune cells into $Rag2^{-/-}IL\text{-}2r\gamma c^{-/-}$ dams. **b**, Representative contour plot. **c**, Immune cell phenotypes of cells used for AT into $Rag2^{-/-}IL\text{-}2r\gamma c^{-/-}$, isolated from age- and gestational day-matched wt female mice. **d-e**, Number of MMc phenotypes in the brain of MMc^{low+AT} compared to MMc^{low} and MMc^{pos} offspring, quantified by flow cytometry at E18.5 (B cells, MMc^{pos} *n*=21, MMc^{low} *n*=21, MMc^{low+AT} *n*=23; T cells, MMc^{pos} *n*=21, MMc^{low} *n*=23, MMc^{low+AT} *n*=24; Microglia, MMc^{pos} *n*=21, MMc^{low} *n*=23, MMc^{low+AT} *n*=24; H-$2D^{b/b}$ cells, MMc^{pos} *n*=21, MMc^{low} *n*=21, MMc^{low+AT} *n*=23) and P8 (B cells, MMc^{pos} *n*=16, MMc^{low} *n*=22, MMc^{low+AT} *n*=9; T cells, MMc^{pos} *n*=16, MMc^{low} *n*=22, MMc^{low+AT} *n*=11; Microglia, MMc^{pos} *n*=15, MMc^{low} *n*=22, MMc^{low+AT} *n*=10; H-$2D^{b/b}$ cells, MMc^{pos} *n*=15, MMc^{low} *n*=20, MMc^{low+AT} *n*=10). **f**, Spectra of absolute power of discontinuous oscillations averaged from all investigated MMc^{pos}, MMc^{low}, and MMc^{low+AT} mice. **g-i**, Numbers of amplitude (MMc^{pos} *n*=10; MMc^{low} *n*=11; MMc^{low+AT} *n*=6) as well as power within θ (MMc^{pos} *n*=11; MMc^{low} *n*=13; MMc^{low+AT} *n*=6), and β (MMc^{pos} *n*=10; MMc^{low} *n*=11; MMc^{low+AT} *n*=6) band of oscillations detected in PL of P8 mice. **j-m**, same as **f-i** for HP (Amplitude, MMc^{pos} *n*=10; MMc^{low} *n*=11; MMc^{low+AT} *n*=5; θ (MMc^{pos} *n*=9; MMc^{low} *n*=13; MMc^{low+AT} *n*=5; β, MMc^{pos} *n*=10; MMc^{low} *n*=13; MMc^{low+AT} *n*=5). Significance was determined by a Kruskal-Wallis one-way ANOVA followed by Dunn's multiple comparison test, $*p < 0.05$, $**p < 0.01$, $****p < 0.0001$. Data in **f** and **j** are displayed as median ± 1.5 IQR. Data in **a**, **d, e**, **g-i**, **k-m** are displayed as mean ± SEM and each circle corresponds to one pup.

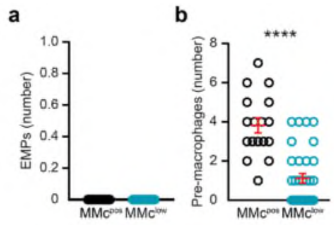

Supplementary Fig. 12. MMc effects on microglia progenitor cells in the yolk sac. **a**, Number of erythro-myeloid progenitors (EMPs) in single yolk sacs from MMc^{pos} (n=19) vs. MMc^{low} (n=33) offspring at E9.5. **b**, same as **a** but for pre-macrophages (MMc^{pos} n=17; MMc^{low} n=33). Significance was determined by a two-sided Mann-Whitney U test, ****$p < 0.0001$. Data are displayed as mean ± SEM and each circle corresponds to one yolk sac.

References cited in the supplement

1 Zhou, Y. *et al.* Human and mouse single-nucleus transcriptomics reveal TREM2-dependent and TREM2-independent cellular responses in Alzheimer's disease. *Nature medicine* **26**, 131-142 (2020).

2 Stuart, T. *et al.* Comprehensive Integration of Single-Cell Data. *Cell* **177**, 1888-1902 e1821, doi:10.1016/j.cell.2019.05.031 (2019).

3 McDavid, A., Finak, G. & Yajima, M. MAST: model-based analysis of single cell transcriptomics. *Genome Biol* **16**, 278 (2015).

4 Livak, K. J. & Schmittgen, T. D. Analysis of relative gene expression data using real-time quantitative PCR and the 2− ΔΔCT method. *methods* **25**, 402-408 (2001).

5 Rodents, F. W. G. o. R. o. G. f. H. M. o. *et al.* FELASA recommendations for the health monitoring of mouse, rat, hamster, guinea pig and rabbit colonies in breeding and experimental units. *Laboratory animals* **48**, 178-192 (2014).

6 Chini, M. *et al.* Resolving and Rescuing Developmental Miswiring in a Mouse Model of Cognitive Impairment. *Neuron* **105**, 60-74 e67, doi:10.1016/j.neuron.2019.09.042 (2020).

7 Bellesi, M. *et al.* Sleep Loss Promotes Astrocytic Phagocytosis and Microglial Activation in Mouse Cerebral Cortex. *J Neurosci* **37**, 5263-5273, doi:10.1523/JNEUROSCI.3981-16.2017 (2017).

8 Matsumoto, K. *et al.* Advanced CUBIC tissue clearing for whole-organ cell profiling. *Nat Protoc* **14**, 3506-3537, doi:10.1038/s41596-019-0240-9 (2019).

9 Voigt, F. F. *et al.* The mesoSPIM initiative: open-source light-sheet microscopes for imaging cleared tissue. *Nat Methods* **16**, 1105-1108, doi:10.1038/s41592-019-0554-0 (2019).

10 Horl, D. *et al.* BigStitcher: reconstructing high-resolution image datasets of cleared and expanded samples. *Nat Methods* **16**, 870-874, doi:10.1038/s41592-019-0501-0 (2019).

11 Dobin, A. *et al.* STAR: ultrafast universal RNA-seq aligner. *Bioinformatics* **29**, 15-21, doi:10.1093/bioinformatics/bts635 (2013).

12 Love, M. I., Huber, W. & Anders, S. Moderated estimation of fold change and dispersion for RNA-seq data with DESeq2. *Genome Biol* **15**, 550, doi:10.1186/s13059-014-0550-8 (2014).

13 Krebs, C. F. *et al.* Autoimmune Renal Disease Is Exacerbated by S1P-Receptor-1-Dependent Intestinal Th17 Cell Migration to the Kidney. *Immunity* **45**, 1078-1092, doi:10.1016/j.immuni.2016.10.020 (2016).

14 Lamprecht, P. *et al.* Changes in the composition of the upper respiratory tract microbial community in granulomatosis with polyangiitis. *J Autoimmun* **97**, 29-39 (2019).

15 O'Sullivan, D. M. *et al.* An inter-laboratory study to investigate the impact of the bioinformatics component on microbiome analysis using mock communities. *Sci Rep-Uk* **11**, 1-14 (2021).

16 Klindworth, A. *et al.* Evaluation of general 16S ribosomal RNA gene PCR primers for classical and next-generation sequencing-based diversity studies. *Nucleic acids research* **41**, e1-e1 (2013).
