# Supplementary material for: Pregnancy-induced maternal microchimerism shapes neurodevelopment and behavior in mice
Source: Nat Commun. 2022 Aug 5;13:4571. doi: 10.1038/s41467-022-32230-2 (PMC9356013; doi:10.1038/s41467-022-32230-2)
Supplement: Supplementary file 4 — Description to Additional Supplementary Information [file 41467_2022_32230_MOESM4_ESM.pdf]

## Description of Additional Supplementary Files

**Supplementary Movie 1**. Tdtomato+ MMc in the offspring's brain. Utilizing a whole organ clearing pipeline, we located tdTomato+ MMc mainly in the PFC and HP at E18.5, but also in other limbic areas, the cerebellum and – albeit sparsely – in other brain regions.
