# Supplementary material for: Pregnancy-induced maternal microchimerism shapes neurodevelopment and behavior in mice
Source: Nat Commun. 2022 Aug 5;13:4571. doi: 10.1038/s41467-022-32230-2 (PMC9356013; doi:10.1038/s41467-022-32230-2)
Supplement: Supplementary file 6 — Reporting Summary [file 41467_2022_32230_MOESM6_ESM.pdf]

nature portfolio

Corresponding author(s): Petra Arck

Last updated by author(s): Jul 6, 2022

# Reporting Summary

Nature Portfolio wishes to improve the reproducibility of the work that we publish. This form provides structure for consistency and transparency in reporting. For further information on Nature Portfolio policies, see our Editorial Policies and the Editorial Policy Checklist.

## Statistics

For all statistical analyses, confirm that the following items are present in the figure legend, table legend, main text, or Methods section.

| n/a | Confirmed | |
|---|---|---|
| ☐ | ☒ | The exact sample size (*n*) for each experimental group/condition, given as a discrete number and unit of measurement |
| ☐ | ☒ | A statement on whether measurements were taken from distinct samples or whether the same sample was measured repeatedly |
| ☐ | ☒ | The statistical test(s) used AND whether they are one- or two-sided<br>*Only common tests should be described solely by name; describe more complex techniques in the Methods section.* |
| ☒ | ☐ | A description of all covariates tested |
| ☐ | ☒ | A description of any assumptions or corrections, such as tests of normality and adjustment for multiple comparisons |
| ☐ | ☒ | A full description of the statistical parameters including central tendency (e.g. means) or other basic estimates (e.g. regression coefficient) AND variation (e.g. standard deviation) or associated estimates of uncertainty (e.g. confidence intervals) |
| ☐ | ☒ | For null hypothesis testing, the test statistic (e.g. *F*, *t*, *r*) with confidence intervals, effect sizes, degrees of freedom and *P* value noted<br>*Give P values as exact values whenever suitable.* |
| ☒ | ☐ | For Bayesian analysis, information on the choice of priors and Markov chain Monte Carlo settings |
| ☒ | ☐ | For hierarchical and complex designs, identification of the appropriate level for tests and full reporting of outcomes |
| ☒ | ☐ | Estimates of effect sizes (e.g. Cohen's *d*, Pearson's *r*), indicating how they were calculated |

*Our web collection on statistics for biologists contains articles on many of the points above.*

## Software and code

Policy information about availability of computer code

| | |
|---|---|
| Data collection | Brain cells were quantified using a LSR Fortessa II (BD Life Sciences) and cells were sorted using the FACSAria III sorter (BD Life Sciences) with the Diva Software (v.9). In vivo electrophysiological data were collected using Digital Lynx SX multichannel extracellular amplifier (Neuralynx) with the Cheetah acquisition software (v.6, Neuralynx). Brain slices were imaged with a Leica DMI6000 confocal microscope. Ultrasonic vocalizations were recorded using an Avisoft UltraSoundGate recording interface. Behavioral experiments were recorded using the Videos Mot2 software (TSE Systems). |
| Data analysis | GraphPad Prism v.8, FlowJo v.9, FIJI Image J v.2.0, and custom scripts written in MATLAB R2017b (MathWorks, USA). Transcriptome and scRNA-sequencing data were analyzed using R. Transcriptome data were analyzed with DESeq and scRNA-sequencing data with Seurat R package (version 3.1.4) as well as MAST R package (version 1.10.0). |

For manuscripts utilizing custom algorithms or software that are central to the research but not yet described in published literature, software must be made available to editors and reviewers. We strongly encourage code deposition in a community repository (e.g. GitHub). See the Nature Portfolio guidelines for submitting code & software for further information.

## Data

Policy information about availability of data

All manuscripts must include a data availability statement. This statement should provide the following information, where applicable:

- Accession codes, unique identifiers, or web links for publicly available datasets
- A description of any restrictions on data availability
- For clinical datasets or third party data, please ensure that the statement adheres to our policy

All data are available at the following open-access repository:

# Field-specific reporting

Please select the one below that is the best fit for your research. If you are not sure, read the appropriate sections before making your selection.

☒ Life sciences ☐ Behavioural & social sciences ☐ Ecological, evolutionary & environmental sciences

For a reference copy of the document with all sections, see nature.com/documents/nr-reporting-summary-flat.pdf

# Life sciences study design

All studies must disclose on these points even when the disclosure is negative.

| | |
|---|---|
| Sample size | Sample sizes were determined based on similar experiments carried out by the groups of the supervising authors (Stelzer, I.A., Urbschat, C., Schepanski, S. et al. Vertically transferred maternal immune cells promote neonatal immunity against early life infections. Nat Commun 12, 4706 (2021). https://doi.org/10.1038/s41467-021-24719-z or Xu, X., Song, L., Kringel, R. et al. Developmental decrease of entorhinal-hippocampal communication in immune-challenged DISC1 knockdown mice. Nat Commun 12, 6810 (2021). https://doi.org/10.1038/s41467-021-27114-w). When experiments had not previously been carried out, we used sample sizes from experiments of similar design. |
| Data exclusions | Data points were excluded if data were >90th and <10th percentile. For scRNA-seq analyses, cells with higher nGene values were excluded (For MMc, nGene cutoff = 5000). We further removed low-quality cells with more than 10% mitochondrial genes of all detected genes. |
| Replication | All experiments besides the scRNA-seq experiments were independently repeated at least three times, while all attempts at replication were successful. Prior to scRNA-seq, the FACS-based enrichment of MMc from the fetal brain was performed and tested more then 5 times. Due to the highly enriched MMc isolated from the fetal brain, we can confirm that the replication were successful. |
| Randomization | We used WT and transgenic mice for timed pregnancies, which were allocated to specific groups based on their genotype. Pregnant female mice (Rag2-/-IL-2rγc-/-) were randomly split into +/- adoptive transfer groups. |
| Blinding | Scoring of behavioral experiments was done in a blinded fashion. The acquisition of all experiments involving MMc-pos and MMc-low animals, the investigators were not blinded, because the reduced number of MMc in the offspring's brain as well as the altered LFP signals do not allow for blinding. Therefore, we decided to repeat the analyses of electrophysiological recordings by a blinded investigator resulting into same results as by non-blinded ones. |

# Reporting for specific materials, systems and methods

We require information from authors about some types of materials, experimental systems and methods used in many studies. Here, indicate whether each material, system or method listed is relevant to your study. If you are not sure if a list item applies to your research, read the appropriate section before selecting a response.

## Materials & experimental systems

| n/a | Involved in the study | |
|---|---|---|
| ☐ | ☒ | Antibodies |
| ☒ | ☐ | Eukaryotic cell lines |
| ☒ | ☐ | Palaeontology and archaeology |
| ☐ | ☒ | Animals and other organisms |
| ☒ | ☐ | Human research participants |
| ☒ | ☐ | Clinical data |
| ☒ | ☐ | Dual use research of concern |

## Methods

| n/a | Involved in the study | |
|---|---|---|
| ☒ | ☐ | ChIP-seq |
| ☐ | ☒ | Flow cytometry |
| ☒ | ☐ | MRI-based neuroimaging |

## Antibodies

| | |
|---|---|
| Antibodies used | Flow cytometry:<br>APC anti-H-2Kd/d/H-2Dd/d (1:400, Biolegend, Cat# 114714), FITC anti-CD45.1 (1:400, Biolegend, Cat# 110705), APC-Cy7 anti-CD45.2 (1:100, Biolegend, Cat# 109823), PE anti-H-2Db/b (1:20, Biolegend, Cat# 111507), AF647 anti-H-2Dd/d (1:200, Biolegend, Cat# 114712), V500 anti-CD11b (1:200, BD, Cat# 562128), PE-Cy7 anti-CD11b (1:200, BD, Cat# 552850), AF700 anti-CD45R/B220 (1:100, ThermoFischer, Cat# 56-0452-80), BV650 anti-CD45R/B220 (1:100, Biolegend, Cat# 103241), PE-Texas Red anti-CD3ε (1:200, ThermoFischer, Cat# 61-0031-82), BV785 anti-CD11c (1:100, Biolegend, Cat# 117335), BV650 anti-Ki-67 (1:100, BD, Cat# 563757),<br><br>Immunohistochemistry:<br>Anti-Iba-1 (1:500, Wako Pure Chemical, Cat# 019-19741) and anti-Vglut1 (1:1000, Millipore, Cat# AB5905), anti-Vglut2 (1:500, Synaptic Systems, Cat# 135404), AF488 goat-anti-guinea pig (1:500, Invitrogen, Cat# A-11073), AF568 donkey-anti-rabbit (1:500, Invitrogen, Cat# A-10042), Hoechst33258 (1:5000, Sigma-Aldrich, Cat# 94403), anti-MAP2 (1:1000, Sigma-Aldrich, Cat# M1406-100UL), AF488 donkey-anti-mouse (1:300, Dianova, Cat# 715-546-150). |
| Validation | We thoroughly describe the protocol for the usage of antibodies in our manuscript. All antibodies were validated by the respective |

Validation

manufacturer, as indicated in the specific data sheets. Each lot of antibody is quality control tested by immunofluorescent staining with flow cytometric analysis by the supplier. They have been previously utilized in Stelzer, I.A., Urbschat, C., Schepanski, S. et al. Vertically transferred maternal immune cells promote neonatal immunity against early life infections. Nat Commun 12, 4706 (2021). https://doi.org/10.1038/s41467-021-24719-z. For quality control, we titrate each antibody to determine its dilution for optimal performance for each application, and repeat its titration after receipt of each new lot.

## Animals and other organisms

Policy information about studies involving animals; ARRIVE guidelines recommended for reporting animal research

| | |
|---|---|
| Laboratory animals | B6.129-Rag2tm1CgnIl2rgtm1Cgn and Balb/c Rag2-/-IL-2rγc-/- were obtained from the institutional animal facility. CByJ.SJL(B6)-Ptprca/J were initially obtained from The Jackson Laboratory (stock number: 006584) and wt CD57BL/6 from Charles River Laboratories. Mice were single-housed (males) or maintained in groups (females) in the animal facility of University Medical Center Hamburg-Eppendorf with regular chow and water provided ad libitum in a normal 12-hour light/12-hour dark cycle at a room temperature of 21°C and controlled humidity at 43%. Experiments were performed using 8-10-week-old females. Males were used for mating from fertile age up until 1 year of age. Females between 9-10 weeks were used in order to generate the utilized offspring. |
| Wild animals | Our study did not involve wild animals. |
| Field-collected samples | Our study did not involve field-collected samples. |
| Ethics oversight | All procedures were approved by the University Medical Center Hamburg-Eppendorf institution-al guidelines and institutional animal welfare officer. The procedures were conform to the re-quirements of the German Animal Welfare Act. Approvals were obtained from the State Authori-ty of Hamburg (Behörde für Justiz und Verbraucherschutz, Amt für Verbraucherschutz, Lebens-mittelsicherheit und Veterinärwesen), Germany (G17/049, N18/111, ORG_927, ORG_1005). |

Note that full information on the approval of the study protocol must also be provided in the manuscript.

## Flow Cytometry

### Plots

Confirm that:

☒ The axis labels state the marker and fluorochrome used (e.g. CD4-FITC).

☒ The axis scales are clearly visible. Include numbers along axes only for bottom left plot of group (a 'group' is an analysis of identical markers).

☒ All plots are contour plots with outliers or pseudocolor plots.

☒ A numerical value for number of cells or percentage (with statistics) is provided.

### Methodology

| | |
|---|---|
| Sample preparation | For the flow cytometry and FACS experiments, we used single cell suspensions derived from E18.5, P8, and P60 brains. Detailed sample processing is described in the Methods section. |
| Instrument | LSR Fortessa (BD Life Sciences) and FACSAria III sorter (BD Life Sciences). |
| Software | Data were acquired in the Diva software and analyzed using FlowJo software (v.9). |
| Cell population abundance | Using LSR Fortessa flow cytometer, 300,000 leukocytes were acquired. For scRNA-sequencing experiments 10,000 maternal microchimeric cells were isolated. |
| Gating strategy | Gates were set manually by using fluorescence minus one (FMO) controls, which are provided in the extended data. Gating strategy is based on a published article and mentioned in the Methods section (Stelzer, I.A., Urbschat, C., Schepanski, S. et al. Vertically transferred maternal immune cells promote neonatal immunity against early life infections. Nat Commun 12, 4706 (2021). https://doi.org/10.1038/s41467-021-24719-z). |

☒ Tick this box to confirm that a figure exemplifying the gating strategy is provided in the Supplementary Information.
